# Network Proteomics: From Protein Structure to Protein-Protein Interaction

**DOI:** 10.1155/2017/8929613

**Published:** 2017-02-16

**Authors:** Guang Hu, Luisa Di Paola, Filippo Pullara, Zhongjie Liang, Intawat Nookaew

**Affiliations:** ^1^Center for Systems Biology, School of Electronic and Information Engineering, Soochow University, Suzhou 215006, China; ^2^Unit of Chemical-Physics Fundamentals in Chemical Engineering, Department of Engineering, Università Campus Bio-Medico, Rome, Italy; ^3^Department of Computational and Systems Biology, University of Pittsburgh, Pittsburgh, PA, USA; ^4^Department of Biomedical Informatics, College of Medicine, University of Arkansas for Medical Sciences, Little Rock, AR, USA

With the emergence of high-throughput “omics” data, network theory is being increasingly used to analyze biomolecular systems. In particular, proteins rarely act alone, which prompted the arrival of proteomics. “Network proteomics” is just defined as the research area that introduces network theory to investigate biological networks ranging from protein structure networks to protein-protein interaction networks. In addition, the application of network proteomics in biomedical fields has increased significantly. This special issue has collected contributions, not only focusing on the state of the art of methodology in “network proteomics” itself but also focusing on the current status and future direction of their applications in translational medical informatics.

This issue starts with discussing the role of protein structure that participates in interactions. N. Raethong et al. developed a strategy to annotate* Aspergillus oryzae*, which would enhance its metabolic network reconstruction. By using the molecular dynamics simulation and principle component analysis, H. Wan et al. investigated the interaction between the last half repeat in TAL effectors and its binding DNA. This work would give a deeper understanding of the recognition mechanism of protein-DNA interactions.

Protein complexes offer detailed structural characteristics of protein-protein interactions. H. Zhang et al. proposed a method by combining the high frequency modes of Gaussian network model and Gaussian Naive Bayes to identify hot spots, which are residues that contribute largely to protein-protein interaction energy. G. Hu et al. performed a comparative study of elastic network model and protein contact network for protein complexes in case of hemoglobin.

Protein-protein interactions not only are limited to binary associations but also employ special topological structures to perform their biological functions. N. Bernabò et al. approached the comparison of two networks models obtained from two different text mining tools to suggest that actin dynamics affect spermatozoa postejaculatory life. J. Xu et al. also compared the topologies of network modes of drug-kinase interactions between established targets and those of clinical trial ones.

Both protein structures and protein-protein interactions are involved in the subject of a growing number of pharmacological studies. F. Ye et al. identified that compounds DC_C11 and DC_C66 are two small inhibitors against protein-protein interactions between CARM1 and its substrates, while Y. Wang et al. identified that CID_70128824, CID_70127147, and CID_70126881 are three potential inhibitors for targeting the androgen receptor to treat prostate cancer. Finally, a review paper completes the issue. X. Li et al. introduced recent advances in the development of network models of identifying synergistic drug combinations.

Altogether, we wish this issue has given a wider development of structural biology and systems biology with the advantage of biological network analysis as well as prospecting the future of this area towards translational bioinformatics and systems pharmacology.

*Guang Hu* *Luisa Di Paola* *Filippo Pullara* *Zhongjie Liang* *Intawat Nookaew*

